# Is the Actual Cautery Beneficial in Ringbone or Not?

**Published:** 1898-04

**Authors:** Charles Bland

**Affiliations:** Roxborough, Pa.


### IS THE ACTUAL CAUTERY BENEFICIAL IN RINGBONE OR NOT?

By Charles Bland, V.S.,

Roxborough, Pa.

I have considered this subject for several years, and have come to the conclusion the horse can be cured of ringbone without the use of the firing-iron, provided you can impress upon the owner the necessity of rest for the animal; but if you cannot, by all means fire him.

I find it very hard matter, in the locality where I practice, to get rest for a horse, unless I have him in my stable and remove his shoes. My treatment is to cut the hair off, and apply hyd. chl. con., dr. j to aq. oz xvi., well rubbed in twice a day for a week, then wash with hot water; after three days repeat the treatment; continue this for a month, and in most cases I succeed in curing the lameness. As you are aware, there are many cases that cannot be cured at all, no matter what treatment you use.

[1] Read at the annual meeting of the Pennsylvania State Veterinary Medical Association, March, 1898.

You must remember an animal should not be put to hard work for several weeks after, and as horses are rising in price, owners will not mind giving us more time for treatment.

Another point: in case you do not cure you have not blemished the horse for the owner to everlastingly tell you and his friends about it.

No doubt many here will say the application is not strong enough; but I am satisfied we have been using too energetic means very often.

I have tried this treatment on spavins, and have relieved a number of animals, and cured some. When I began, and for several years after, I fired and blistered, but seldom do now.

